# Language teaching with video-based technologies: Creativity and CALL teacher education

**DOI:** 10.3389/fpsyg.2022.995652

**Published:** 2022-09-02

**Authors:** Hongbo Song, Ziwei Liu

**Affiliations:** School of Foreign Languages, Wuhan University of Science and Technology, Wuhan, China

**Keywords:** video-based technologies, machinima, teacher education, language teaching, immersive learning

Ever since the emergence of Web 2.0, computer-assisted language learning (CALL) has afforded abundant opportunities for language learners to experience real and diversified language situations (Chun et al., 2016), and it has also facilitated language learners' self-directed learning (Reinders and White, 2011). Thus far ample research has been conducted to document how video-based technologies boost students' language learning motivation, self-confidence, interest in language learning tasks, and language gains (e.g., González-Lloret and Ortega, 2014; Cai et al., 2022). However, what is yet to be investigated is how language learning affordances emerge from machinima, which is defined, in a language learning context, as teachers' or learners' self-created digital videos that involve learners in “an immersive environment such as a three-dimensional (3D) digital game or immersive virtual world” (Thomas and Schneider, 2021, p. 7). What has been even less examined in the current literature is how and to what extent language teachers can benefit from CALL teacher education programs on the production and application of machinima in language teaching classrooms. That said, the good news is that these gaps have newly been addressed in the book hereby under review, i.e., *Language Teaching with Video-Based Technologies* (Thomas and Schneider, 2021).

Consisting of six chapters, the book is committed to exploring the potential of video-based digital technologies in promoting language learning. Specifically, it examines the role of machinima, which is used as pedagogical tools in language classrooms for learners to conduct authentic language learning collaboratively and interactively. In addition, the book provides illuminating insights as to how language teacher education programs can be designed to maximize the potential of machinima in language learning.

The introduction of the book details the goals of teacher education in a CALL context and gives a brief account of the development of digital video and machinima. As claimed by the authors, the goal of CALL teacher education is not to equip teachers with skills of using a specific piece of software. Rather it purports to enable them: (1) to understand how technology, theory, and pedagogy are closely interwoven and inseparable; and (2) to identify and comprehend the factors that affect the effective integration of technologies into specific language teaching contexts. In analyzing the role of digital video and machinima in language teaching, the chapter specifically highlights how a video-based approach to language learning/teaching fosters learners' collaborative and intercultural competence, and how it cultivates their interdisciplinary knowledge.

Chapter 2, entitled “Immersive Education and Machinima”, provides a critical review of the existing research on immersive learning environments, video-based learning, and machinima production in educational settings. The chapter demonstrates the generally positive effects of immersive learning in virtual environments on students' language learning, where they can conduct autonomous, collaborative, and project-based learning. It also discusses the advantages of machinima production such as cost and time effectiveness, and its disadvantages in relation to technical challenges, video quality, and lack of non-verbal cues in comparison to real-life filmmaking. The authors contend at the end of the chapter that the integration of machinima into language classrooms can increase students' engagement levels and reduce their inhibition in the language learning process.

Chapter 3, “Technology-Mediated Project-Based Language Teaching”, probes into the origins of project-based language learning in task-based language teaching approaches and identifies its core characteristics which focus on real-world activities guided by socio-constructivism and communicative language teaching. The use of digital technologies can be used to address some limitations of both approaches (Ellis, 2009) and extend project-based learning through the innovative use of video and machinima production. By means of immersive environments in foreign language learning contexts and merger with the principles of content and language integrated learning (CLIL), project-based learning approaches provide opportunities for scaffolded learning that is collaborative and communicative in nature and deals with real-world problems in highly motivating, learner-centered ways.

The ensuing Chapter 4, entitled “Creating and Field Testing Machinima in the Language Classroom”, evaluates ways that machinima can enhance language learning. This is exhibited in five pilot studies in various settings: (1) using machinima in a commercial learning context in Germany; (2) teaching ESP (English for specific purposes) and general English in the Czech Republic; (3) understanding mathematics with machinima and CLIL in the Netherlands; (4) piloting machinima with learners of Turkish; and (5) using machinima in a military university in Poland. In these studies, data were collected from teachers and students who applied machinima in their classrooms and studies; and for that matter, both quantitative and qualitative methods were used, including questionnaires, classroom observations, interviews and focus group discussions. The main findings reveal that the question of whether students learn more effectively with machinima is rather complex and involves many factors. Overall, the results appear quite positive—The majority of teachers displayed enthusiasm about teaching with ready-made machinima and 75% of the students expressed comfort about the learning experience with machinima. Whilst machinima is justified for allowing learners to reflect on their language habits and behaviors, it is also criticized for its characters' unnatural facial expressions and gestures.

In Chapter 5, “Evaluating a Machinima CALL Teacher Education Course”, the authors analyze trainee feedback on the two CALL teacher education courses. Data were collected by means of questionnaires, interviews, and focus group discussions, which vividly illustrate how the surveyed teachers experienced the courses, how their digital literacy skills developed, and how they gained an understanding of machinima. The research results demonstrate that the trainee teachers understood the value of creating and using machinima in their professional environment and were able to apply their newly acquired skills to everyday teaching. They also proved competent in achieving the intended goals: learning new skills, enhancing language learning with machinima, nurturing interest and motivation in the classroom through machinima, working collaboratively, and stimulating learners' passion for learning.

The last chapter, i.e., Chapter 6, summarizes the methodology employed in the research project and the main findings of the research. Notably, the chapter points out future directions of research on video-based approaches to language teaching and learning. They include but are not limited to: (1) adopting ethnographic approaches to better capture learners' and teachers' behaviors in immersive virtual environments; (2) incorporating more learner perspectives in studying the effectiveness of using machinima in immersive virtual environments; (3) devoting more efforts to examining how CALL teachers cultivate their general digital skill sets and efficiently employ technological tools to augment language learning.

Overall, the book *Language Teaching with Video-Based Technologies* contributes greatly to our understanding of CALL in general and the role of machinima in language teaching/learning in particular. By sketching out informative reviews of research on immersive education, technology-mediated project-based language teaching, and machinima, the authors, first and foremost, empower readers to catch a quick glimpse of the theoretical underpinnings of these approaches to language teaching/learning (e.g., socio-constructivist learning, community of practice). Furthermore, the reviews in the book promise to motivate language teachers or learners to experiment with these approaches in their own teaching or learning, where they can verify if these approaches are applicable to their contexts and how different contextual and individual factors interweave in a complex manner. Then they may get impelled to further study what modifications and adaptations are needed to better take advantage of these approaches to facilitate language learning processes. In addition, the insights and implications rendered by the CALL teacher education project in the book will also inspire language teacher educators to design and tailor their training materials based on contextual factors.

Besides, the book is also helpful for researchers and language teaching practitioners to conduct situated CALL research for its methodological guidelines and directions for future research (Chapters 4 and 6). With the book, they should be able to better understand the nature of CALL and become more adept in using different kinds of technologies to aid their teaching and enhance students' learning experience.

Admittedly, the book is not without shortcomings. One faultiness, for example, lies in the inconsistency of terms used throughout the book, such as “immersive virtual environment” (Page 16) vis-à-vis “virtual immersive environments” (Page 186). The writing in the book would appear more rigorous if terms like these remained consistent with each other.

As language teaching researchers in China, we warmly welcome such a book introducing innovative approaches to language teaching and learning. However, whether machinima applies to the Chinese context remains to be seen and explored. To our knowledge, little empirical research has documented how machinima could be effectively integrated into language classrooms in China. In a culture that places much emphasis on preparing students to pass various examinations (Reynolds and Teng, 2021), and one that has long been implementing knowledge-based language teaching (Xu and Long, 2020), we believe the research methodologies advocated in the monograph are insightful and inspiring for language teachers in China to bring machinima into their classrooms *via* doing relevant research. But our Chinese colleagues may find it worthwhile to go a step further to successfully integrate machinima into their classroom teaching. They may have to equip themselves with practitioner research methodologies (Ellis, 2012) unmentioned in the book by Thomas and Schneider (2021), such as action research (Burns, 2009) and exploratory research (Allwright, 2003). These methodologies will help teachers conduct local research in their classrooms and empower them to seek a solution (Ellis, 2012) when they encounter difficulties in striking a balance between an examination-oriented curriculum and the innovative teaching approach using machinima as a pedagogical tool.

Transforming the long-standing knowledge-based language teaching into a kind that is, at least partially, communicative/interactional competence-based is bound to be a tough way to go and requires language teachers and teacher educators to exercise their agency (Xu and Long, 2020). However, the way is worth trying given the positive research findings (e.g., improved learner motivation, interest, passion, etc.) reported by Thomas and Schneider (2021).

Another insight gleaned from the book that may be especially beneficial for language teachers in China is the necessity to conduct teacher training/education with regard to how to create machinima and use it in classrooms, and how to implement needs analysis before introducing machinima into language teaching. As is reported in the book, teachers who participate in the teacher training project are more likely to change the way they teach in physical classrooms, and to “involve their students in a form of co-participation in the process of learning with machinima” (Thomas and Schneider, 2021, p. 184). This is what language teachers in China need for transforming their traditional classrooms and for fostering their students' communicative competence and the spirit of collaboration.

Based on all the above discussed, we, therefore, believe this monograph will immensely benefit teachers, researchers, and students in applied linguistics, particularly those relevant to CALL as an innovative approach to language teaching and learning.

## Author contributions

ZL wrote the initial draft of the article. HS provided several rounds of critical and constructive feedback to the drafts, and added some more comments and details in the process of writing. The final draft is the result of ZL and HS's collective effort. All authors contributed to the article and approved the submitted version.

## Funding

This article was supported by: (1) The Hubei Provincial Key Research Project for Philosophy and Social Sciences in Higher Learning Institutions (Grant Number: 21D008) and (2) The Incubation Project of High-level Humanities and Social Sciences of Wuhan University of Science and Technology (Grant Number: W201907).

## Conflict of interest

The authors declare that the research was conducted in the absence of any commercial or financial relationships that could be construed as a potential conflict of interest.

## Publisher's note

All claims expressed in this article are solely those of the authors and do not necessarily represent those of their affiliated organizations, or those of the publisher, the editors and the reviewers. Any product that may be evaluated in this article, or claim that may be made by its manufacturer, is not guaranteed or endorsed by the publisher.
